# Clinical Course of Ophthalmic Findings and Potential Influence Factors of Herpesvirus Infections: 18 Month Follow-Up of a Closed Herd of Lipizzaners

**DOI:** 10.1371/journal.pone.0079888

**Published:** 2013-11-20

**Authors:** James O. Rushton, Jolanta Kolodziejek, Alexander Tichy, Norbert Nowotny, Barbara Nell

**Affiliations:** 1 Clinic for Surgery, Dentistry and Ophthalmology, Department of Companion Animals and Horses, University of Veterinary Medicine Vienna, Vienna, Austria; 2 Viral Zoonoses, Emerging and Vector-Borne Infections Group, Institute of Virology, Department of Pathobiology, University of Veterinary Medicine Vienna, Vienna, Austria; 3 Institute of Population Genetics, Department of Biomedical Sciences, University of Veterinary Medicine Vienna, Vienna, Austria; 4 Department of Microbiology and Immunology, College of Medicine and Health Sciences, Sultan Qaboos University, Muscat, Oman; Justus-Liebig-University Giessen, Germany

## Abstract

**Background:**

To date the influence of herpesviruses on the development of equine ocular diseases has not been clearly determined.

**Objective:**

The purpose of this study was to illustrate the course of equine ocular findings over a period of 18 months at 6 month intervals, in correlation with the results of herpesvirus detection.

**Methods:**

266 Lipizzaners in 3 federal states of Austria underwent complete ophthalmologic examination 4 times. Blood samples, nasal- and conjunctival swabs were obtained at the same time and used for the detection of the equid gammaherpesviruses EHV-2 and EHV-5 using consensus herpesvirus PCR and type-specific qPCRs. Ophthalmic findings and results of herpesvirus PCRs were recorded and statistically analysed using one-way ANOVA, and multiple logistic regression analysis to determine the influence of herpesvirus infections and other contributing factors on the presence of ophthalmic findings.

**Results:**

In the first, second, third and fourth examination period 266, 261, 249 and 230 horses were included, respectively. Ophthalmic findings consistent with herpesvirus infections included conjunctival- and corneal pathologies. Statistical analysis revealed that the probability of positive herpesvirus PCR results decreased with progressing age; however the presence of corneal findings increased over time. At the time of each examination 45.1%, 41.8%, 43.0%, and 57.0% of horses with conjunctival or corneal findings, respectively, were positive for EHV-2 and/or EHV-5. However, 31.6%, 17.6%, 20.1%, and 13.0% of clinically sound horses were positive for these herpesviruses at each examination period, too.

**Conclusion:**

Based on the results of our study there is a significant influence of young age on EHV-2 and/or EHV-5 infection. Corneal pathologies increased over time and with progressing age. Whether the identified findings were caused by herpesviruses could not be unequivocally determined.

## Introduction

Since the recovery of the first gammaherpesvirus of horses, equid herpesvirus 2 (EHV-2) from a foal with respiratory disease in 1962, continuous research on EHVs has been undertaken [1]. Today a plethora of publications on the detection of EHVs is available [2]–[11]. In most of these studies, the influence of EHV-2, and EHV-5 on ophthalmic diseases, specifically conjunctivitis and keratitis, was studied [7], [10], [11], the results of which show that there is no direct association between clinical findings and the detection of herpesviruses (HVs). Experimental research, however, has shown that immunosuppression with systemic steroids and infection with EHV-2 results in keratoconjunctivitis and upper respiratory tract diseases [3]. This indicates that immunodeficiency is an immanent factor for HVs to cause clinical symptoms. In the publication by Fetsch et al. [6], the immune status of sound horses and horses suffering from keratoconjunctivitis was determined. The results of that study indicated that clinically sound EHV-2 positive horses had decreased levels of B-lymphocytes.

Most studies on the determination of the influence of HVs on ocular findings are set up in a similar way. A group of horses with findings characteristic of virally induced keratitis are compared with a group of sound horses and tested once for the presence of HV DNA [7], [8], [10], [11]. The animals are usually owned by clients, under various boarding systems, with varying levels of performance and other confounding factors. It was therefore the purpose of this study to determine the ocular health status and the prevalence of HV infection in a large study population of a single horse breed, with limited variation in boarding systems, feeding, and performance. The study population was examined and sampled at regular intervals over a period of 18 months; all potential “stress” factors were noted and included in the statistical analysis. The aim of the study was to establish the influence of HVs on ophthalmic findings, with regard to additional contributing factors.

## Materials and Methods

### Ethics Statement

The study was part of an installed routine monitoring program, all procedures were approved by officials of the Spanish Riding School and the Federal stud Piber. The ethics committee of the University of Veterinary Medicine Vienna was informed and approved of the study protocol. No official permit was required, since all diagnostic and therapeutic interventions were aimed to benefit the health of the study population. Conjunctival swabs were obtained under topical anaesthesia to minimize irritation. All efforts were made to minimize any suffering.

### Study Population

All Lipizzaners at 3 locations [federal state stud Piber (located in the Austrian federal state of Styria), Heldenberg (Lower Austria) and the Spanish Riding School in Vienna], born before the 1^st^ of January 2011 were included in the study.

### Ophthalmic Examination

Horses were subjected to complete ophthalmic examination 4 times over a period of 18 months, at 6 month intervals. They were examined using slit lamp biomicroscopy (Kowa portable slit-lamp SL-14, CR Medical, Linz, Austria) as well as direct and indirect ophthalmoscopy (Heine Omega 2C of Heine Optotechnik GmbH & Co KG, Hersching, Germany) following induced mydriasis (Mydriatikum “Agepha”, Vienna, Austria) as described by Rushton et al. [14]. Ophthalmic findings were documented on eye examination sheets. EDTA blood as well as nasal cytobrush swabs (NS) and conjunctival cytobrush swabs (CS) from both eyes were collected under topical anaesthesia (Novain 0.4% “Agepha”, Vienna, Austria). If horses could not be examined or sampled by manual constraint only, they were excluded from the study.

### Sample Obtainment

Nasal- and conjunctival swabs were taken using sterile cytobrushes (CELLETTATM brush cell collector of Engelbrecht Medizin- und Labortechnik GmBH, Edermünde, Germany), kept in tubes with 0.5 ml virus isolation medium and stored at −80°C. Blood samples (2 ml) were collected in sterile EDTA-containing vacutainer tubes (VACUETTE K3 EDTA of Greiner Bio-One GmbH, Kremsmünster, Austria). EDTA blood was centrifuged at 4300×g for 10 min in a cooled centrifuge. The buffy coat was obtained; incubated in erythrocyte lysis buffer (EL-buffer, Qiagen, CA, USA) for 10 min and centrifuged. This step was repeated twice, the obtained PBMC pellet was digested with Proteinase K (Qiagen, Valencia, CA, USA) and tissue lysis buffer (ATL-buffer, Qiagen, CA, USA) at 55°C for 8 h.

### Nucleic Acid Extraction

The entire PBMC lysate and 140 µl of each conjunctival- and nasal swab material were used for nucleic acid extraction. The process was performed using QIAamp viral RNA mini kit (Qiagen, Valencia, CA, USA) according to the manufacturer’s instructions. Despite its name this kit allows extraction of total nucleic acid from the samples. The nucleic acid extracts were stored at −80°C.

All samples were tested individually by nested consensus HV PCR, as well as specific EHV-5 and EHV-2 qPCRs.

### Consensus HV PCR (Including Nested PCR), EHV-1 and -4 PCR and Gel Electrophoresis

The nested consensus HV PCR protocol was established by Van Devanter et al. [13] and was modified by Rushton et al. [14], employing a commercial Fast Cycling PCR Kit (Qiagen, USA). Due to its extremely wide detection range, which includes almost all members of the *Herpesviridae* family, this protocol has been used by many researchers to amplify a highly conserved region within the DNA polymerase gene of HVs.

Primers used for EHV-1 & -4 PCR (in one reaction tube) were EHV-1+4 F 5′-GTACCAGCGTTACCGTSTCAA-3′, EHV-1 R 5′-GGCGTCATATACCACTGTGTCC-3′, and EHV-4 R 5′-GCTACTCCGCATGTTGACTA-3′. The primers - originating from the dissertation by Posch [15] - are located within the glycoprotein 13 (gp 13) gene. PCR was carried out using Fast Cycling PCR kit (Qiagen, Valencia, CA, USA). The concentrations of master mix reagents were identical to consensus PCR. Activation of DNA polymerase took place at 96°C for 5 min, followed by 45 cycles of amplification using the following PCR profile: denaturation at 95° for 25 sec, annealing at 55°C for 5 sec and primer extension at 68°C for 25 sec, completed by polymerisation at 72°C for 1 min.

Gel electrophoresis was performed as described by Rushton et al. [14].

### EHV-5 and EHV-2 Realtime, Quantitative PCR (qPCR)

EHV-5 and EHV-2 qPCRs were conducted as described by Rushton et al. [14] using a QuantiFast Probe PCR kit (Qiagen, Valencia, CA, USA).

Both realtime PCRs were applied as qualitative assays (negative/positive). Ct values below 40 were considered positive.

### Sequencing

Nucleotide sequencing was carried out on amplification products of the nested consensus HV PCR, where the corresponding EHV-2- and EHV-5 specific qPCRs were negative. These amplicons were sequenced as described previously [14]. Sequencing of PCR products was performed in an ABI PRISM 310 Genetic Analyser (PerkinElmer, Waltham, MA, USA).

### Sequence Alignment

All sequences were compared with each other using the software program Align Plus Program (Scientific & Educational Software, Version 3.0, serial no. 43071) and compared to the corresponding HV sequences in GenBank using BLAST (http://blast.ncbi.nlm.nih.gov/Blast.cgi).

### Statistical Analysis

Statistical analysis was performed using SPSS v19 (SPSS Inc., Chicago, Illinois, USA). The influence of HV infection, and potential influence factors such as gender, location, housing situation (open box, closed box, free stall) on ophthalmic findings, was determined using cross tables, and tested for significance using Chi^2^-Test. Horses positive for EHV-2 and EHV-5 were compared with negative horses regarding age, the difference was tested for significance using Student’s T-Test. To determine the influence of positive results for HV and respective findings multiple logistic regression models were used. A combination of various influence factors (boarding, location, transfer from one location to another and the level of performance) with positive HV results on the presence of ophthalmic findings was determined with descriptive predictability models. In order to determine a delayed ocular inflammatory response due to the presence of HVs, pattern recognition analysis was performed. A P-value <0.05 was considered statistically significant.

## Results

### Study Population

In the first, second, third, and fourth examination period 266, 261, 249, and 230 horses were included, respectively. A total of 36 horses were lost to follow-up, either due to sale, death, or progressing uncooperative behaviour. The study population was distributed among Piber, Vienna, and Heldenberg with 172 (64.7%), 72 (27.1%), and 22 (8.3%) horses at the first, 156 (59.8%), 67 (25.7%), and 38 (14.6%) at the second, 148 (59.4%), 68 (27.3%), and 33 (13.3%) at the third and 128 (55.7%), 65 (28.3%), and 37 (16.1%) horses at the fourth examination, respectively. The gender distribution consisted of mares, stallions and geldings with 114 (42.9%), 146 (54.9%), and 6 (2.3%) at the first, 114 (43.7%), 141 (54.0%), and 6 (2.3%) at the second, 109 (43.8%), 134 (53.8%), and 6 (2.4%) at the third, and 102 (44.3%), 123 (53.5%), and 5 (2.2%) at the fourth study period. Mares and geldings were kept exclusively in Piber, stallions were distributed throughout all 3 locations. The mean ages of the study population for each examination period were 5.6 years (SD ±6.3), 6.5 years (SD ±6.3), 7.2 years (SD ±6.3), and 8.7 years (SD ±6.2), respectively.

Three types of boarding systems are used to accommodate the study population, to wit, open boxes in Vienna and Heldenberg, closed boxes in Vienna, Heldenberg, and Piber, together with freestalls in Piber. Detailed descriptions of the boarding systems are outlined in the recent publication by Rushton et al. [12]. Fourteen (5.3%), 50 (19.2%), 47 (18.9%), and 49 (21.3%) horses were housed in open boxes in the first, second, third, and fourth study period. Freestalls were the boarding system for 147 (55.3%), 135 (51.7%), 113 (45.4%) and 102 (44.3%) horses for each examination period. The remainder were housed in closed boxes.

### Ophthalmic Findings

**Figure 1:**
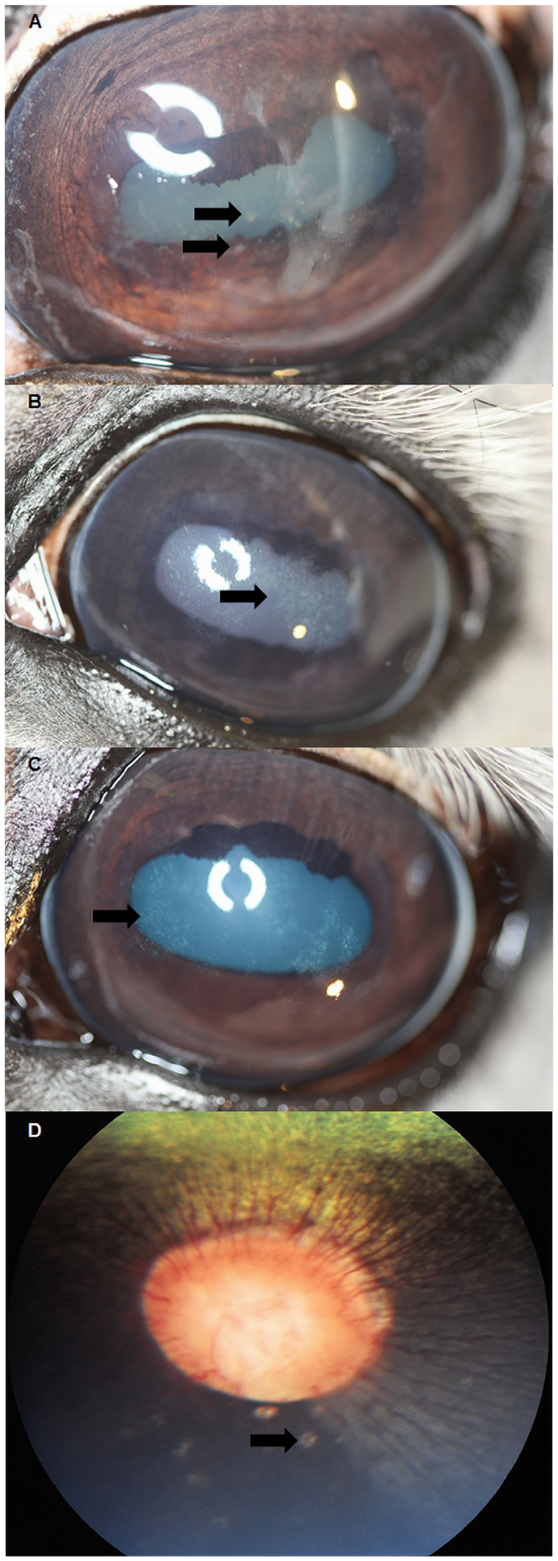
Ophthalmic findings of inflammatory origin. A: Photograph of stallion diagnosed with superficial punctate keratitis with associated corneal oedema. Note the arrow pointing to superficial punctate infiltrates. B & C: Photograph of stallions diagnosed with anterior stromal opacities, suggestive of antecedent corneal inflammation. Note the arrows pointing towards the geographic areas of anterior stromal opacity. D: Fundus photograph of a stallion diagnosed with “bullet-hole” lesions in the peripapillary area. Note the arrows pointing towards the peripapillary “bullet-hole” lesions.

**Figure 2:**
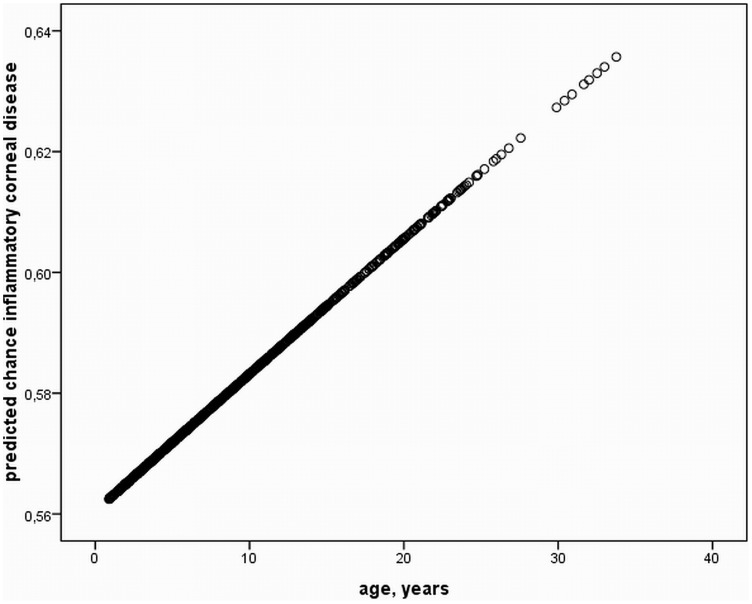
Prediction of inflammatory corneal disease. Predicted chance of inflammatory corneal disease according to age based on T-Test analysis over the entire study period. A significant increase of corneal diseases of inflammatory origin exists (P<0.05).

**Figure 3:**
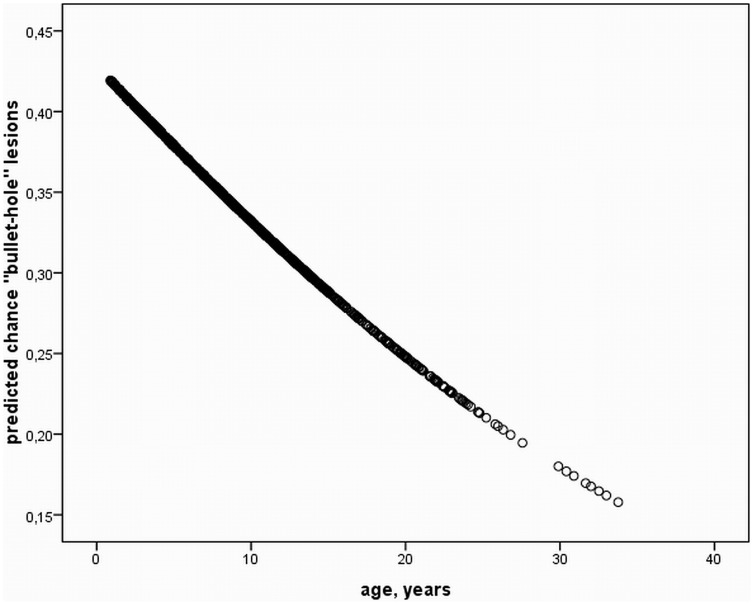
Prediction of inflammatory fundus disease. Predicted chance of “bullet-hole” lesions according to age based on T-Test analysis over the entire study period. A significant decrease of “bullet-hole” lesions with increasing age exists (P<0.05).

A plethora of non-inflammatory (non-vision threatening), and findings of suspected inflammatory origin (potentially vision threatening) were identified in the study population. This paper is focused on findings consistent with suspected inflammatory origin, to determine any influence of HV infection on the presence of respective findings. The remaining non-inflammatory, mostly congenital ophthalmic findings are described in detail in the recent publication by Rushton et al. [12]. Ophthalmic findings of inflammatory origin consisted of conjunctivitis, inflammatory corneal diseases and “bullet-hole” as well as “butterfly” lesions. There were no cases of acute or chronic uveitis in any horse at any examination period. Regarding conjunctivitis 118 (44.4%), 145 (55.6%), 151 (60.6%), and 170 (73.9%) cases were identified in each consecutive examination period. Corneal findings of suspected inflammatory origin consisted of superficial punctate keratitis (**Figure 1A**), anterior stromal keratitis (**Figure 1B & C**), and ghost vessels. One or several of these findings were identified in 53 (19.9%), 67 (25.7%), 83 (33.3%), and 83 (36.1%) horses in the first, second, third, and fourth examination, respectively. More specifically anterior stromal keratitis was found in 43 (16.2%), 48 (18.4%), 52 (20.9%), and 53 (23.0%); superficial punctate keratitis in 16 (6.0%), 15 (5.7%), 19 (7.6%), and 7 (3.0%). Ghost vessels were identified in 7 (2.6%), 14 (5.4%), 28 (11.3%), and 37 (16.1%) horses for each study period. As for retinal findings of potential inflammatory origin “bullet-hole” lesions (**Figure 1D**) were found in 79 (29.7%), 87 (33.3%), 91 (36.5%), and 90 (39.1%) horses in each respective period. “Butterfly” lesions were identified in 1 horse in the first 2 examinations and in 2 horses in the third and fourth exam. The influence of age on the existence of each ophthalmic finding revealed a statistically significant difference between mean ages of horses positive vs. negative for inflammatory corneal diseases (p = 0.005) (**Figure 2**), and more specifically anterior stromal keratitis (p<0.001), as well as “bullet-hole” lesions (p<0.001) (**Figure 3**). Corneal findings of suspected inflammatory origin were identified in significantly older horses with a mean age of 9.7 years (SD ±6.8), whereas “bullet-hole” lesions were found in younger horses with a mean age of 7.8 years (SD ±4.9). The remaining findings were not significantly influenced by age, by means of Student’s T-Test.

Chi^2^-Test of potential individual influence factors on ophthalmic findings revealed a significant influence of location on the presence of conjunctivitis (p<0.001) and “bullet-hole” lesions (p = 0.029) over the entire study period. Both findings were significantly more often identified in horses stabled in freestalls in Piber. Furthermore significantly fewer horses were identified with “bullet-hole” lesions in the third and fourth examination period (p = 0.026; p = 0.029 respectively). The remainder of influence factors, such as transfer from one location to another and training did not contribute significantly to the presence of ophthalmic findings when analysed individually.

### EHV-1 & -4 PCR

All samples positive on consensus HV PCR, but negative in both EHV-2- and EHV-5 specific qPCRs were tested for EHV-1 & -4, despite the fact that the horses in all locations were routinely vaccinated against these viruses. Forty-eight (18.0%) samples from the initial sample obtainment met these criteria, all were negative for EHV-1 & -4, respectively.

### Cumulated Results of qPCR and Sequence Analysis

The results of both EHV-2, and EHV-5 qPCR, as well as sequence analysis were summarised for statistical analysis. A total of 31 (11.7%) horses were positive for EHV-5 in the first study period, 51 (19.5%) in the second, 39 (15.7%) in the third, and 65 (28.3%) in the fourth. EHV-2 was positive in 62 (23.3%), 30 (11.5%), 69 (27.7%), and 34 (14.8%) cases. Double infection of both EHV-2 & -5 was detected in 105 (39.5%), 68 (26.1%), 42 (16.9%), and 58 (25.2%) horses. Nasal swabs were positive most often, followed by conjunctival swabs, and PBMCs in last place. The results of the initial examination period – published by Rushton et al. [14] - differ from the ones described in this publication, due to differences in methods of statistical analysis, however the number of horses identified with HVs remains the same.

**Figure 4:**
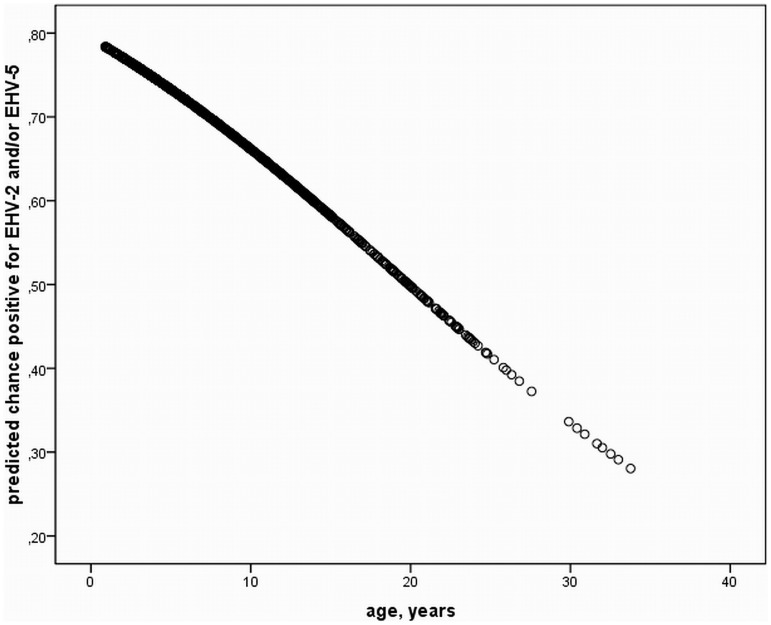
Prediction of positive herpesvirus results. Predicted chance of positive PCR results for EHV-2 and/or EHV-5 according to age based on T-Test analysis over the entire study period. A significant decrease of samples positive for herpesviruses with increasing age exists (P<0.05).

A statistically significant influence of regional distribution on positive HV results was identified. Horses stabled in Piber were positive for EHV-5 and/or EHV-2 significantly more often than in Vienna, and Heldenberg (p<0.001). Significantly more horses in freestalls were positive for EHV-5 and/or EHV-2 at every examination period (p<0.001). Comparison of mean ages of horses positive vs. negative for HVs using T-test revealed a statistically significant influence of young age on positive PCR results (**Figure 4**). The mean age for horses positive for EHV-5 was 6.9 years (SD ±6.0), for EHV-2 7.5 years (SD ±6.3), and EHV-2 & -5 double infection 5.5 years (SD ±5.3).

### Influence of Positive EHV-2 and/or EHV-5 PCR Results on Ophthalmic Findings

To illustrate the influence of PCR results on ophthalmic findings, all positive HV PCR results, regardless whether EHV-2, or -5, or both were summarised and compared with the results of ophthalmic findings, of suspected inflammatory origin. Due to the lack of knowledge of the aetiogenesis of “bullet-hole” lesions, these were included in the summary of ophthalmic findings, to determine a potential influence of EHV-2 and/or -5 on the pathogenesis. When “bullet-hole” lesions were added to conjunctivitis and corneal findings of suspected inflammatory origin 138 (51.9%), 121 (46.4%), 122 (49.0%), and 144 (62.6%) horses with ophthalmic findings also had HV infections. The number of horses with conjunctivitis, and corneal findings of suspected inflammatory origin, which were positive for HVs on PCR, was 120 (45.1%), 109 (41.8%), 107 (43.0%), and 131 (57.0%) for the first, second, third, and fourth examination period. Horses with no ophthalmic abnormalities were positive for HVs in 84 (31.6%), 46 (17.6%), 50 (20.1%), and 30 (13.0%) for each respective examination.

In order to determine a delayed inflammatory response of the equine eye due to positive HV PCR results, which had been obtained 6 months earlier, pattern recognition analysis was performed. If inflammatory corneal diseases, conjunctivitis and “bullet-hole” lesions were summarised, 34 (13.0%) horses with new findings were identified between the first and second, 18 (7.2%) between the second and third, and 24 (10.4%) between the third and fourth examination period. Analysis of inflammatory corneal diseases and conjunctivitis with positive HV PCR results revealed 44 (16.9%), 22 (8.8%), and 32 (13.9%) horses with new findings in the same time frame. If only inflammatory corneal diseases were considered, 21 (8.0%), 24 (9.6%), and 23 (10.0%) horses were identified with new findings.

### Influence of Co-factors and PCR Results on Ophthalmic Findings

Using multiple logistic regression models the predictability for the development of ophthalmic findings based on location, transfer from one location to another, boarding system, training, and HV PCR results, was determined for each time period and over the whole study period. No notable influence of any combination of co-factors and PCR results on the development of ocular findings of suspected inflammatory origin was determined.

A table summarizing the entire study including all potential influence factors, ophthalmic findings and results of herpesvirus PCRs is presented as supporting information (Table S1).

## Discussion

Based on the results of this extensive, long term study a number of statements may be expressed, regarding both the prevalence of ophthalmic findings and the current state of HV infection in a large study population of a single horse breed. Regarding ophthalmic findings, conjunctivitis is most prevalent in horses boarded in freestalls. No other influencing factors apart from the location and housing system could be determined within the scope of this study. Conjunctivitis is a symptom, which is associated with several aetiologies, both infectious and non-infectious. It is a subjective finding and largely dependent on the examiner’s experience, unless clinically overt ocular discharge and blepharospasm are present. In this study moderate to severe redness and chemosis of the conjunctiva were termed conjunctivitis. In some cases ocular discharge and mild blepharospasm were also present, however no distinction between grades of conjunctivitis was made for statistical analysis. To the author’s knowledge an influence of the boarding system on the presence of conjunctivitis has never been described in the literature. A likely explanation for this observation is that mainly young horses are kept in a confined space, with close contact to one-another. Dominance behaviour and dynamics in hierarchy may contribute to the level of stress the horses experience. Infectious agents may spread to locally immunosuppressed conjunctival tissue, thus resulting in conjunctivitis. Based on the results of statistical analysis, however, the influence of EHV-2, and EHV-5 on conjunctivitis could not be established. Other infectious agents were not the subject of this study and therefore not investigated further. Similar results, regarding the association of conjunctivitis and detection of HVs, were retrieved in 2 publications and a recent dissertation [8], [11], [16].

Horses with corneal findings, especially anterior stromal opacities, due to suspected antecedent corneal inflammation increased in number throughout the study period and a significant influence of age on the presence of these findings was determined. This is a novel finding in the literature, probably due to the lack of long term follow-up studies of large study populations.

“Bullet-hole” lesions, which represent remnants of antecedent focal chorioretinitis [17] were prevalent at rates of 30–39%, reaching their maximum number of horses in the age group below 7.8 years. They are considered to be incidental findings in otherwise sound horses and are non-vision threatening, if less than 20 lesions are found in a single eye. According to the literature the aetiology has been associated with EHV-1 infection at an early age [17]. In the first examination and sample obtainment, samples which were positive on consensus HV PCR but negative on EHV-2 and EHV-5 qPCR, were also tested for EHV-1 & -4 using a duplex PCR. All samples were negative for both HVs. This was the likely result of an effective vaccination program, which had been installed in reaction to the disastrous EHV-1 disease of 1983 in the Federal state stud Piber, which led to the abortion of 40 foetuses and the death of 10 mares due to progressive neurological disease [18], [19]. “Bullet-hole” lesions were associated with stabling in Piber and were significantly less prevalent in the final 2 examination periods if horses were boarded in open boxes. This observation may be explained by the regional distribution of boarding systems. The population in Piber mainly consists of young colts and mares. The significant influence of young age on the presence of “bullet-hole” lesions may also explain the significant association between this clinical finding and the location. Open boxes are mainly found in Vienna and Heldenberg, inhabited by older horses, thus there was a negative correlation between this finding and that particular boarding system. However the fact, that “bullet-hole” lesions were significantly more often identified in younger horses is novel, according to the authors’ knowledge. The potential influence of EHV-2 and/or -5 on the development of “bullet-hole” lesions was tested, due to the lack of knowledge on the pathomechanism of this finding. Due to the size of the study population and the long term follow-up, a trend of increasing numbers of horses with this finding, due to the presence of EHV-2 and/or EHV-5 would have likely been noticed.

The prevalence of EHV-2 and/or EHV-5 was significantly influenced by the age of the animals included, reaching the highest rates of infection in horses younger than 5.5, 6.9, and 7.5 years for EHV-2 & -5, EHV-5, and EHV-2, respectively. This finding is corroborated by a study from 2010 by Marenzoni et al., which determined a clear association between the prevalence of EHV-5 and young age [9]. That particular study was also performed in a large population of Lipizzaners, in Italy. To the author’s knowledge this is the first report of a significant influence of young age on the presence of EHV-2 and EHV-2 & -5 double infections in consecutive examinations over an 18 month period.

Regarding the influence of results of HV PCR on ophthalmic findings, rates of more than 50% of horses with ocular findings of suspected inflammatory origin were positive for HVs. Horses with no findings were positive for EHV-2, and -5 at rates of less than 31%. This difference is marked but not statistically significant over all 4 examination periods. The infection rate of horses with ocular findings, which were positive for HVs is higher than described in the literature. In a recent dissertation 4.6% of horses with ocular findings were positive for EHV-2, 31.3% for EHV-5 and 12.2% for both viruses [16]. The study population was of equal size to the present study. The distribution of positive results for HV PCR in horses with keratoconjunctivitis in another study was 34.8%, 6.5% and 4.2% for EHV-5, EHV-2, and EHV-5 & -2 double infections, respectively [11]. Potential reasons for the higher infection rates of horses in our study may be the mean age of the study population (5.3–7.5 years), which was lower than in the study by Richter et al. (10 years) [11]. The mean age of the study population in the dissertation by Sonderegger was not specified, but is likely to have been higher than in the present study, since mainly client owned horses were used [16].

A novel finding of this study is the rate of eye diseases with regard to HV PCR results of 6 months prior to the examination. However, due to the limited average rate of horses with newly developed ocular findings of suspected inflammatory origin, we were not able to test for significance or determine any additional contributing factors. To the authors’ knowledge, this is the first study to consider additional factors, such as the level of performance, transfer from one location to another or boarding system on the development of suspected HV-associated ocular findings. Using multiple logistic regression models the percentage of predictability for each combination of several potential contributing factors was calculated. However none of the combinations of factors exceeded a predictability of more than 50%. We therefore cannot make any predictions for the development of ocular findings beyond the scope of this study, based on the factors, which were analysed. As with pattern recognition analysis, testing for significance was not an option for this statistical method.

With this long term study, we were able to gather more information on time-related dynamics of the prevalence of equid herpesviruses and acquired ocular diseases, which would not have been possible based on a single examination [14]. We were also able to confirm some of the observations made in the initial examination – influence of young age and location on the presence of equid gammaherpesviruses over 3 consecutive follow-ups.

Despite a plethora of advantages of this study - i.e. a large population of a single horse breed in a well-managed environment under close observation for a duration of 18 months, which were examined and sampled by the same examiners - there were some limitations. The main ophthalmologic findings identified were not, for the most part, acute inflammations of the respective locations, but remnants of antecedent disease. The fact that horses developed corneal opacities, consistent with prior inflammation within a period of 6 months with no obvious signs of ocular discomfort, suggests that most ocular pathologies are acquired subclinically. Analysis of samples at the acute onset of corneal pathologies would probably have influenced the results of this study. Furthermore PCR detection methods do not allow any conclusions on the infectivity of the detected virus. In general, diagnosis of EHV-2/−5 infections may be performed using 3 methods. Indirect methods of detection include serology, which was first described by Kono and Kobayashi in 1964 [20], but due to several shortcomings can still not be considered a standardized method. Secondly cell culture isolation attempts, using rabbit kidney- or equine-derived cells such as equine kidney or equine dermis cells, which will pick up (only) infectious virus by developing a cytopathic effect. However, the cytopathic effect of EHV-2 becomes usually visible only after several blind passages 12–21 days p.i. [21]. EHV-5 may be isolated on rabbit kidney-13, equine fetal kidney or Vero cells and is reported to induce cytopathic effects upon 3–4 passages [22]. Cell culture isolation was attempted with samples positive for asinine herpesvirus 5 (AsHV-5) - an incidental finding in this study and topic of a separate publication -, which is closely related to EHV-5 and -2, however unsuccessfully (unpublished data). Cell culture isolation attempts of EHV-5 and -2 -positive samples were not performed during this study, due to their equally poor quality to grow in cell cultures. Finally DNA amplification, as used here, employing consensus-, duplex-, or qPCRs, are considered the standard methods for virus detection in equid gammaherpesvirus epidemiologic studies [21].

Based on the results of this study, there was no statistical proof of a link between EHV-2, and/or EHV-5-infection, and the presence of ocular findings. The contribution of additional potential “stress-factors”, such as environment or management could not be clearly elucidated. However the rate of infection of horses with ocular findings was higher than previously described in the literature. An additional noteworthy fact is the rate of newly acquired corneal findings between examination periods. Previously the influence of EHV-2 on host immune response has been described, which may predispose horses to secondary infections with other pathogens [23]. Based on this information involvement of secondary pathogens, or an immune-mediated process due to EHV-2 induced immune modulation are likely explanations for the development of these findings. Further research on the host immune response to HVs, rather than the influence of outside factors should therefore be continued.

## Supporting Information

Table S1 **Overview of influence factors, ophthalmic findings and herpesvirus results over 18 months.**
(XLSX)
